# Rapid detection and molecular survey of *bla*VIM, *bla*IMP and *bla*NDM genes among clinical isolates of *Acinetobacter baumannii* using new multiplex real-time PCR and melting curve analysis

**DOI:** 10.1186/s12866-019-1510-y

**Published:** 2019-06-10

**Authors:** Hossein Goudarzi, Elnaz Sadat Mirsamadi, Zohreh Ghalavand, Mojdeh Hakemi Vala, Hamed Mirjalali, Ali Hashemi

**Affiliations:** 1 Department of Microbiology, Faculty of Medicine, Shahid Beheshti University of Medical Sciences, Tehran, Iran; 2 Department of Microbiology, Faculty of Medicine, Tehran Medical Sciences, Islamic Azad University, Tehran, Iran; 3 Foodborne and Waterborne Diseases Research Center, Research Institute for Gastroenterology and Liver Diseases, Shahid Beheshti University of Medical Sciences, Tehran, Iran

**Keywords:** *Acinetobacter baumannii*, Melting curve analysis, Multiplex real-time PCR, Single tube reaction

## Abstract

**Background:**

*Acinetobacter baumannii* is a cosmopolitan bacterium that is frequently reported from hospitalized patients, especially those patients who admitted in the intensive care unit. Recently, multiplex real-time PCR has been introduced for rapid detection of the resistance genes in clinical isolates of bacteria. The current study aimed to develop and evaluate multiplex real-time PCR to detect common resistance genes among clinical isolates of *A. baumannii*.

**Results:**

Multiplex real-time PCR based on melting curve analysis showed different T_m_ corresponding to the amplified fragment consisted of 83.5 °C, 93.3 °C and 89.3 °C for *bla*IMP, *bla*VIM and *bla*NDM, respectively. Results of multiplex real-time PCR showed that the prevalence of *bla*IMP, *bla*VIM and *bla*NDM among the clinical isolates of *A. baumannii* were 5/128(3.9%), 9/128(7.03%) and 0/128(0%), respectively. Multiplex real-time PCR was able to simultaneously identify the resistance genes, while showed 100% concordance with the results of conventional PCR.

**Conclusions:**

The current study showed that *bla*VIM, was the most prevalent MBL gene among the clinical isolates of *A. baumannii* while no amplification of *bla*NDM was seen. Multiplex real-time PCR can be sensitive and reliable technique for rapid detection of resistance genes in clinical isolates.

## Background

*Acinetobacter baumannii* is known as one of the most common bacteria that is frequently found in the hospitalized patients in Intensive Care Unit (ICU) [1, 2]. Several types of infections resulting from *A. baumannii* have been reported, with systemic infection and pneumonia currently the most important forms [3–5]. Currently, several studies have indicated the resistance of *A. baumannii* to the broad spectrum of antibiotics such as beta-lactams [6–8]. Meanwhile, incorrect and imprecise prescription of the drugs leads to the emergence of bacteria that are resistant to more than one class of antibiotics, knowing as multi drug resistant (MDR) and extreme drug resistant (XDR) strains. According to the guidelines, those bacteria resistant to more than three different following classes: carbapenems, aminoglycosides, ampicillin-sulbactam, cephalosporins and flouroquinolones, are known as MDR strains [9–12]. However, emergence of MDR among *A. baumannii* strains, as one of the most important concerns of physicians, decreases the number of drugs of choice, especially in the clinical practice.

Antibiotics from the carbapenems family are recommended as the most effective drugs for treatment of *A. baumannii* infections [6, 13]. However, *A. baumannii* is able to rapidly acquire the carbapenems resistance genes such as Metallo-Beta-Lactamases (MBLs) [14]. Several reports of MBL-producing strains has led to increased numbers of studies on the prevalence as well as designing reliable methods for detection of the most prevalent MBL genes among clinical strains of *A. baumannii* [7, 15, 16]. MBLs-producing strains of *A. baumannii* have been frequently reported in Iran [17–19]. Therefore, laboratory identification of the drug resistance genes is a pivotal step in the early assessment and management of hospital infections due to *A. baumannii*, particularly in ICU patients.

During the past two decades, molecular approaches have been introduced as a useful tools in order to screen antimicrobial resistance genes in different isolates of *A. baumannii* [20–22]. Real-time PCR is a molecular tool that is able to provide more accurate and rapid results in comparison with conventional PCR techniques. Recently, multiplex real-time PCR was applied as a reliable test for immediate and coincident identification of more than one MBL gene in the clinical isolates of bacteria [23–26].

The current study aimed to apply multiplex real-time PCR assay in a single tube for simultaneous identification of three common types of MBL genes (*bla*IMP*, bla*VIM and *bla*NDM), in clinical isolates of *A. baumannii* using the melting curves analysis. To our knowledge, this is the first study that used multiplex real-time PCR in a single tube, for the concurrent identification of *bla*IMP*, bla*VIM and *bla*NDM genes in the clinical isolates of *A. baumannii*.

## Results

In order to perform multiplex real-time PCR, *A. baumannii* isolates were selected from those strains that were previously phenotypically examined for piperacillin-tazobactam, ceftriaxon, ceftazidime, cefepime, imipenem, doripenem-ertapenem, cefotaxime, and ampicillin-sulbactam, using disc diffusion (Mast Co., UK) regarding to CLSI 2013 guidelines. Accordingly, antibiotic susceptibility pattern showed that all isolates were resistant to piperacillin-tazobactam, while 71.1% of isolates were sensitive to ampicillin-sulbactam [20].

**Fig. 1:**
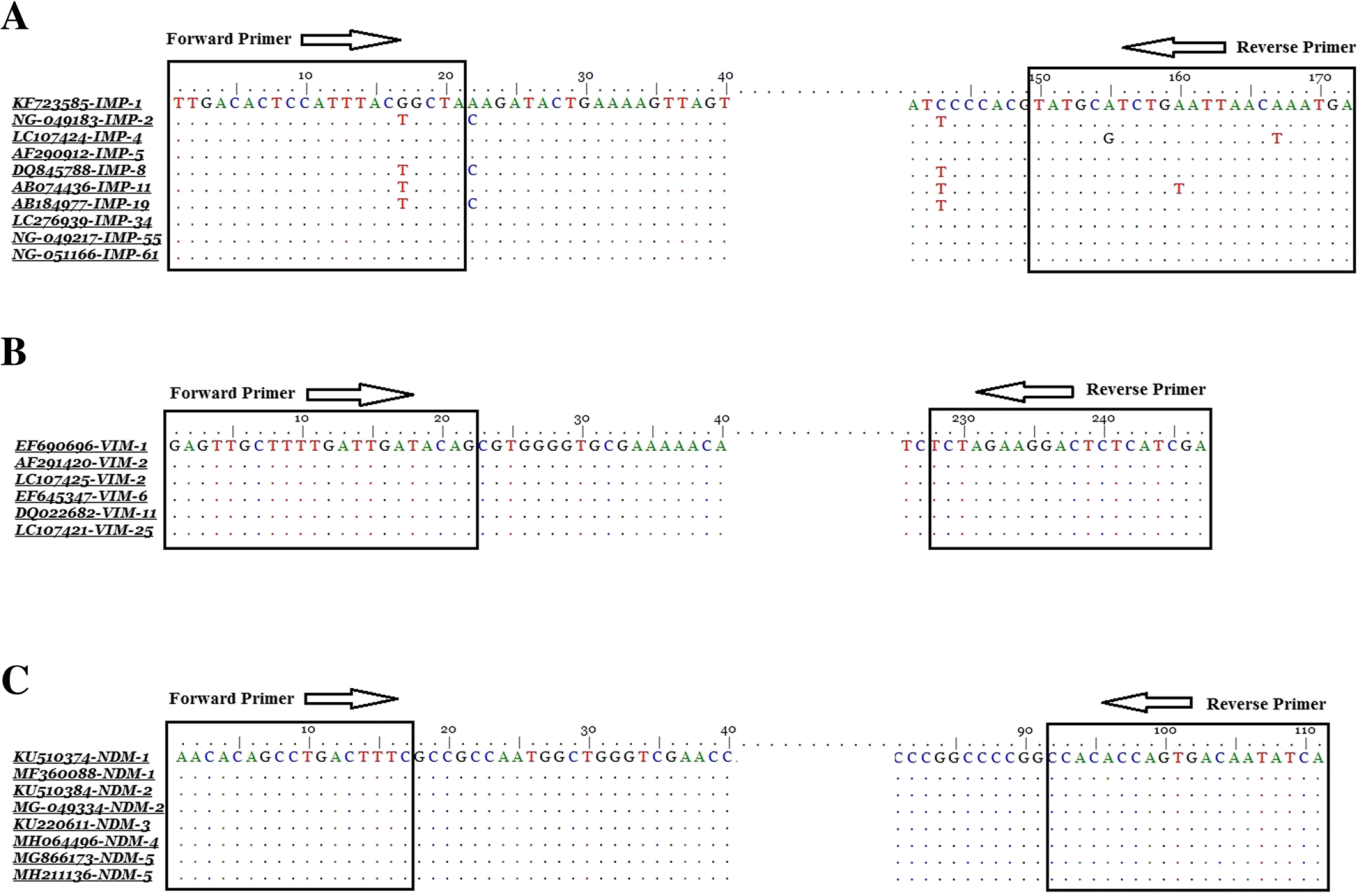
Alignment of some available subtypes of **a**: *bla*IMP, **b**: *bla*VIM and **c**: *bla*NDM using BioEdit software. The position of primers for each gene was specified using box and arrow

**Fig. 2:**
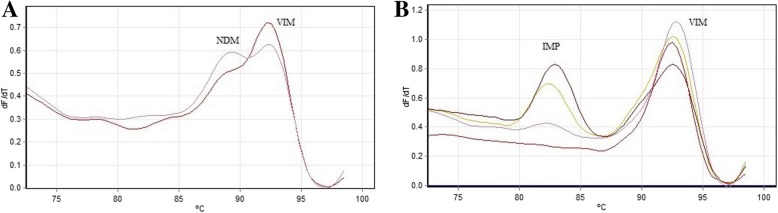
Melting curve analysis of *bla*IMP*, bla*VIM and *bla*NDM. **a** Melting curve analysis of *bla*NDM and *bla*VIM. The purple curve shows amplification of both NDM and VIM genes in a single tube while the dark red curve shows only the VIM peak. **b** Melting curve analysis of *bla*IMP*, bla*VIM in clinical samples. The results show that three of the isolates were positive for both VIM and IMP, while one of the curve shows only VIM peak

**Fig. 3:**
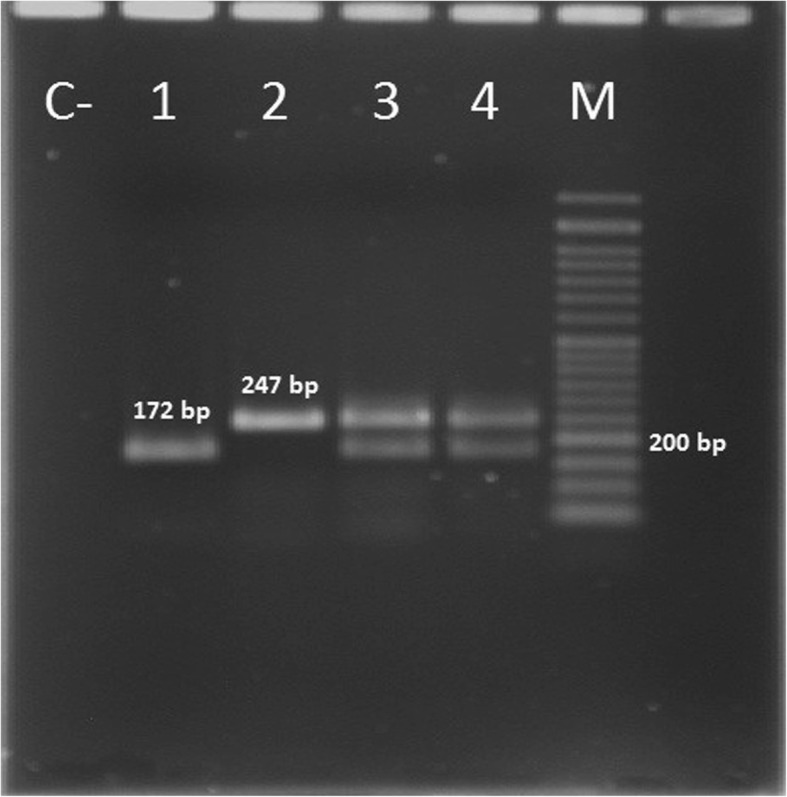
Electrophoresis shows expected fragments on 2% agarose gel that were amplified using multiplex real-time PCR. M: 50 bp ladder; C-: negative control, 1: *bla*IMP (172 bp), 2: *bla*VIM (247 bp), 3 and 4: multiplex amplification of *bla*IMP and *bla*VIM

In silico analysis showed that designed primers amplified all available alleles of the mentioned MBL gene families (Fig. 1). To evaluate the accuracy of the amplified fragment of each gene, multiplex SYBR green real-time PCR was separately conducted on the positive control of *bla*IMP, *bla*VIM and *bla*NDM. Melting curve analysis of each amplicon was generated that showed the fragment T_m_ 83.5 °C, 93.3 °C and 89.3 °C for *bla*IMP, *bla*VIM and *bla*NDM, respectively (Table 1). Multiplex real-time PCR was also performed for all three MBL genes in a single tube and showed discriminative melting temperatures in the melting curve analysis (Fig. 2). For more confirmation, all amplified fragments were electrophoresed on 2% agarose gel and stained with ethidium bromide that showed discriminative bands (Fig. 3). The results of the multiplex real-time PCR showed that all the PCR-positive isolates had the amplification plots and also relevant melting temperatures for the resistance genes. The results of multiplex real-time PCR showed that the prevalence of *bla*IMP, *bla*VIM and *bla*NDM among the clinical isolates of *A. baumannii* were 5/128(3.9%), 9/128(7.03%) and 0/128(0%), respectively. However, the results of antibiotic susceptibility pattern and multiplex real-time PCR were compared in (Table 2).

**Table 1:** Designed primers and fragment T_m_ of the amplified fragments for multiplex real-time PCR

Resistance genes	Primer (5′-3′)	Fragment length (bp)	Fragment melting Temprature (°C)
IMP	F: TTGACACTCCATTTACTGCTA R: TCATTTGTTAATTCAGATGCATA	172	83.5
VIM	F: GAGTTGCTTTTGATTGATACAG R: TCGATGAGAGTCCTTCTAGA	247	93.3
NDM	F: AACACAGCCTGACTTTCG R: TGATATTGTCACTGGTGTGG	111	89.3

**Table 2:** The comparison results of antibiotic susceptibility test and multiplex real-time PCR

Antibiotics	Antibiotic susceptibility test	Multiplex real-time PCR	
	Resistant (high/intermediate) No (%)	*bla*IMP –positive No (%)	*bla*VIM –positive No (%)
Piperacillin-tazobactam	128 (100)	5/128 (3.9)	9/128 (7.03)
Ceftriaxone	128 (100)	5/128 (3.9)	9/128 (7.03)
Ceftazidime	128 (100)	5/128 (3.9)	9/128 (7.03)
Cefepime	126 (98.4)	5/126 (3.97)	9/126 (7.14)
Imipenem	121 (94.53)	5/121 (4.13)	9/121 (7.44)
Doripenem	127 (99.22)	5/127 (3.94)	9/127 (7.09)
Ertapenem	128 (100)	5/128 (3.9)	9/128 (7.03)
Cefotaxime	128 (100)	5/128 (3.9)	9/128 (7.03)
Ampicillin-sulbactam	37 (28.9)	5/37 (13.519)	9/37 (24.32)

## Discussion

*A. baumannii* is a major pathogen in hospitalized patients, particularly in the ICU ward. During the recent years, the increasing reports of the multidrug resistance strains of this bacterium has been highlighted *A. baumannii* as a major public health issue, in the world [27, 28]. Multidrug resistance of *A. baumannii* usually occurs to beta-lactams, aminoglycosides, fluoroquinolones and also carbapenems. Notably, the wide distribution of the resistance strains of this worldwide bacterium to betalactam drugs family has been led to increase of concerns of physicians in the clinical practices for the rapid detection of drug resistance isolates, particularly in the hospitalized patients.

Many studies have suggested molecular techniques to detect and identify carbapenemase genes among the broad spectrum of bacteria [29–31]. However, the conventional molecular methods are often time- and cost-consuming due to their need to the post-PCR analysis. Real-time PCR approaches that target resistance genes including carbapenemase have been frequently applied, so far. Martin-Pena and colleagues developed a TaqMan quantitative real-time PCR to identify resistance genes corresponding to imipenem, ciprofloxacin, colistin and amikacin in clinical isolates of *A. baumannii* [32]. Recently, multiplex real-time PCR was employed for concurrent detection of several resistance genes from one or more than one families. In a study conducted by Mendes in Brazil, multiplex real-time PCR was exploited to detect IMP and VIM types, SPM-1, GIM-1, and SIM-1 in ATCC reference and laboratory strains of gram-negative bacteria consisted of *P. aeruginos*, *Escherichia coli*, *Acinetobacter calcoaceticus*, *Klebsiella pneumonia*, *Neisseria meningitidis*, *Neisseria perflava*, *Neisseria lactamica*, *Neisseria sicca*, *Salmonella serovar typhimurium*, *Enterobacter aerogenes* [33]. In another study performed by Monteiro and colleagues SYBR green real-time PCR followed by the High Resolution Melting (HRM) curve analysis was defined to detect *bla*KPC, *bla*GES, *bla*IMP, *bla*VIM, *bla*OXA-48 and *bla*NDM-1 genes in positive strains of *Enterobacteriaceae* (no.21), *A. baumannii* (no. 1) and *P. aeruginosa* (no. 8) [24]. The main limitation of these studies was this fact that real-time PCR for each resistance gene was conducted in separated tubes but in a single real-time PCR run. In order to overcome this limitation, the primers of the current study were designed to identify resistance genes in a single tube using melting curve analysis based on T_m_ of the amplified fragments.

Zheng in 2013 introduced a duplex TaqMan real-time PCR assay for the concurrent identification of *bla*NDM and *bla*KPC genes in Enterobacteriaceae. According to this study, 7 and 10 isolates carried *bla*NDM and *bla*KPC, respectively [26]. Recently, Yang and colleagues described two sets of multiplex real-time PCR to distinguish *A. baumannii* and Non- *baumannii Acinetobacter* spp., and also detect *bla*NDM, *bla*OXA-23-like, *bla*OXA-40-like, *bla*OXA-51-like, and *bla*OXA-58-like genes in clinical samples using melting curve analysis [34]. In both of these studies the results of multiplex real-time PCR showed 100% concordance with the results of conventional PCR. Our findings were in line with the results of these studies and represented that all isolates that were positive for *bla*IMP and *bla*VIM by conventional PCR, also showed amplification using multiplex real-time PCR.

In the current study, although 100% concordance was seen between the results of conventional PCR and multiplex real-time PCR, the results of disc diffusion showed higher range of antibiotic resistance in comparison with molecular tests. Theoretically, it was suggested that bacteria employ at least three mechanisms against beta-lactams that are including: a) destruction of the antibiotics using beta-lactam enzymes, b) applying efflux pumps to control influx and efflux of antibiotics and c) reducing the access to- and affinity of penicillin-binding protein (PBP) [35, 36]. However, it seems that *A. baumannii*, more likely uses several mechanisms against beta-lactams antibiotics and therefore, both phenotypic and genotypic analysis should be applied to provide more information about the drug resistance patterns.

Furthermore, in the current study, although the genes of *bla*IMP and *bla*VIM were detected in the clinical samples, there was no amplification of *bla*NDM. Our results were in accordance with the study performed by Milillo that showed no amplification of NDM gene in clinical isolates of *Acinetobacter*. As supported by literature, it assumed that the main reason of this observation could be related to the ability of *Acinetobacter* to harbor only one copy of NDM gene [37].

### Limitations

The Ambler class B-metallo-beta-lactamases (MBL) and carbapenem -hydrolysing class D beta-lactamases (CHDLs) are two common mechanisms for the carbapenem resistant in *A. baumannii*. However, since in the current study, only three common types of MBL genes (*bla*IMP*, bla*VIM and *bla*NDM) were investigated, it seems that high negative rate in multiplex real-time PCR results may not reflect the actual rate of resistant genes, particularly CHDL genes in *A. baumannii*.

## Conclusion

Multiplex real-time PCR is a sensitive, rapid and precise method that is able to simultaneously detect the MBL resistance genes among clinical isolates of *A. baumannii*. Furthermore, the current study represented that there was 100% concordance between the result of conventional PCR and multiplex real-time PCR; thus, the later method could be suggested as a rapid and sensitive technique for multiplex detection of the resistance genes among the clinical isolates of bacteria, particularly in emergency cases who are hospitalized in ICU.

## Methods

### Patients and specimens

This study had received ethical approval from the Ethics Committee of the Shahid Beheshti University of Medical Science (SBMU), Tehran, Iran. The current study was performed on samples, which were previously collected by Goudarzi and colleagues [20]. The samples were collected from 128 patients who had been hospitalized in different wards including ICU, Surgery, Nourosurgery, Orthopedics, Infectious, etc. of two hospitals in Tehran.

### DNA extraction

In order to achieve enough *A. baumannii* colonies for DNA extraction, the obtained isolates were incubated overnight at 36 °C in LB medium. Then, each sample was centrifuged at 8000 rpm for 3 min, supernatant was discarded and the pellet was introduced to DynaBioTM Blood/Tissue DNA Extraction Mini Kit (Takapouzist Company, Iran). Purified DNA was kept out in − 20 °C until molecular investigation.

### Primer designing and detection

Primers were designed using the online software Primer-BLAST (http://www.ncbi.nlm.nih.gov/tools/primer-blast/) according to available *bla*IMP, *bla*VIM, *bla*NDM genes in GenBank database with accession numbers: KF723585, GQ288396 and KF951474, respectively. All the primers were checked in silico to amplify all available alleles of the mentioned MBL gene families. The primers amplified fragments with different lengths and melting temperatures to provide enough resolution in the conventional PCR and multiplex real-time PCR, respectively (Table 1). In the case of IMP and VIM, the positive isolates that were sequenced and submitted in GenBank database with accession numbers: KU685506 to KU685508 were used as positive controls. In addition, the positive case of NDM-1 was kindly provided by Dr. Shahcheraghi from Institute Pasteur of Iran. In order to determine specificity of the designed primers, clinical isolates of *A. baumanni* and *Pseudomonas* spp., which were phenotypically and molecularly non- MBLs resistant isolates, were tested using the designed primers. In addition, the isolates were screened for carrying *bla*IMP, *bla*VIM and *bla*NDM genes using conventional PCR [20]. It is neccesary to mention that conventional PCR was performed using the current desined primers.

### Multiplex real-time PCR

Each reaction was accomplished in final volume of 25 μl containing 2X Real-time PCR Mastermix (Bioneer, Korea), 5 pmol of each primer, 1 μl of ROX, 6.5 μl of DEPC water and 2 μl of template using Rotor Gene 6000 (Corbett, Australia) instrument. The reaction mixture was subjected to 95°c, 10 min, 40 Cycles including 95°c, 10 s, 55°c, 37 s, 72°c, hold 20 s ramping from 70 °C to 99 °C at 0.02 °C s^− 1^. Furthermore, melting curve analysis was determined using Rotor Gene 6000 software.

## Data Availability

The data associated with this manuscript consisted of normalized melting curves are included in the article.

## Abbreviations

ICU: Intensive Care Unit
MBLs: Metallo-Beta-Lactamases
MDR: Multi drug resistant
